# Prospective bidirectional associations between depression and chronic kidney diseases

**DOI:** 10.1038/s41598-022-15212-8

**Published:** 2022-06-28

**Authors:** Xiaowei Zheng, Wenyan Wu, Suwen Shen

**Affiliations:** 1 Department of Public Health and Preventive Medicine, Wuxi School of Medicine Jiangnan University, 1800 Lihu Road, Binhu District, Wuxi, 214122 Jiangsu Province China; 2 Institutes of Biology and Medical Sciences, Soochow University, Suzhou, 215000 Jiangsu China; 3 Wuxi Municipal Center for Disease Control and Prevention, Wuxi, 214023 Jiangsu China

**Keywords:** Psychology, Diseases

## Abstract

Previous studies had reported the mutual relation between depression and chronic kidney diseases (CKD). This study aimed to investigate potential bidirectional relationships between depression and CKD. Participants more than 45 years from the China Health and Retirement Longitudinal Study (CHARLS) were included in present study. In study I, we tended to assess the association between baseline depression with the risk of subsequent CKD. In study II, we aimed to examine whether the onset of CKD could predict the development of depression. Multivariate logistic regression models were used to calculate odds ratios (ORs) and 95% confidence intervals (95% CIs) in study I and study II, respectively. In study I, 301 (6.16%) respondents experienced CKD in participants without depression, and 233 (8.48%) respondents experienced CKD in participants with depression. Participants with depression had higher risk of developing CKD with the corresponding ORs (95% CIs) was 1.38(1.08–1.76). In study II, 1333 (22.29%) subjects in the non-CKD group and 97 (27.17%) in CKD group developed depressive symptoms. Individuals with CKD had higher risk of developing depression than those without CKD, with the multivariate ORs (95% CIs) was 1.48(1.23–1.78). Significant bidirectional relationships remained in both sensitivity and subgroup analyses. Findings demonstrate bidirectional relationships between depression and CKD. Individuals with depression were associated with increasing risk of CKD; in addition, CKD patients had higher risk of developing depression.

## Introduction

Clinically relevant depressive symptoms and clinical depression and are very common among older people in the community^1^. Depression is one of the leading cause of years of life lost due to disability (YLD) worldwide and resulted in 43 million years YLD in 2017^2^, which corresponding to 10.3% of the total YLD across all diseases^3^. In China, the reported prevalence of depressive symptoms was approximately 32–37% in 2008–2015 among the middle-aged and elderly adults^4^. Previous studies have explored the effect of depression, alone or as a comorbidity, on overall health status^5–8^.

In recent years, the prevalence of chronic kidney disease (CKD) is increasing in the general population, leading to an increasingly prominent public health problem^9^. According to the Global Burden of Disease (GBD) Study, CKD accounted for 2.2% of all deaths in 2017, and the disability-adjusted life-years (DALY) rate for CKD increased to 1.43% worldwide^2^. In China, the reported overall prevalence of CKD was 10.8% in 2012^10^, and the prevalence of CKD (estimated glomerular filtration rate (eGFR) < 60 mL/min/1.73 m^2^) was 6.5% in 2018^11^. Individuals with CKD have a greatly increased risk of all-cause mortality, cardiovascular mortality, and kidney failure^12,13^.

Several previous studies had reported the mutual association between depression and CKD. Individuals with elevated depressive symptoms tended to had higher risk of renal function decline, new-onset CKD, ESRD, and hospitalization with acute kidney injury^14–16^. While, CKD patients had a higher prevalence of depression than that reported for patients with other chronic diseases^17^, and it is estimated that the prevalence of depression varies from 23 to 46% in patients with ESRD or in dialysis populations^18–20^. Furthermore, depression in CKD had been shown to be associated with multiple poor outcomes^21,22^. Both epidemiologic evidence and shared pathophysiologic mechanisms suggest a potential bidirectional relation between depression and CKD.

In the present study, we hypothesized that there might be a bidirectional association between depression and CKD, and investigated the bidirectional association between depression and CKD based on the data from the China Health and Retirement Longitudinal Study (CHARLS). In study I, we evaluated the longitudinal association of baseline depression with subsequent incident of CKD in follow-up. In study II, we aimed to examine whether the onset of CKD could predict the incident of depression in follow-up.

## Methods

### Study design

The CHARLS study used a multistage clustering sample method to select participants from 28 provinces in mainland China^23^. A total of 17,708 participants from 10,257 households recruited from 28 provinces within China were included at baseline (2011–2012, Wave 1). CHARLS respondents are then followed up every 2 years, using a face-to-face computer-assisted personal interview. Two subsequent follow-ups were carried out in 2013–2014 (Wave 2) and in 2015–2016 (Wave 3), respectively. At each wave, trained personnel conducted face-to-face interviews to collect information including participants’ sociodemographic characteristics, medical history, health behaviors, and measures of general health status and functioning including depressive symptoms. More detailed description of CHARLS can be found in previous publications^23,24^. This study was approved by Biomedical Ethics Review Committee of Peking University, and all participants signed informed consents. The ethics application for collecting data on human subjects in CHARLS was approved by the Biomedical Ethics Review Committee of Peking University (IRB00001052-11,015). All study methods were carried out based on the Declaration of Helsinki.

### Study sample

**Figure 1:**
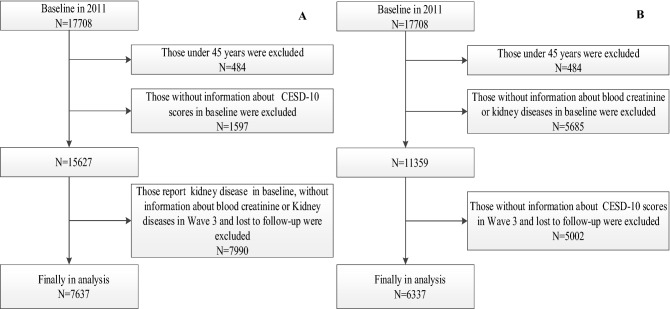
Flow chart of the selection of the study population for Study I (**A**) and Study II (**B**).

In study I, individuals who met all of the following criteria were included: aged at least 45 years, reported information about the Center for Epidemiological Studies Depression Scale-10 (CESD-10) in Wave 1, without CKD diseases in Wave 1 and reported information about CKD in Wave 3. Finally, a total of 7637 individuals were eligible for subsequent analysis (Fig. 1A). In study II, individuals who met all of the following criteria were included: aged at least 45 years, reported information about CKD and in Wave 1, without depression in Wave 1 and reported information about the CESD-10 in Wave 3. Finally, a total of 6337 individuals were eligible for subsequent analysis (Fig. 1B).

### Depression, CKD and covariates assessments

The CESD-10 was administrated to measure depressive symptoms at baseline and each follow-up visit of the CHARLS^25^, which has been proved to be a reliable and valid approach to detect depression in Chinese adults^26,27^. The CESD-10 scale incorporates depressed mood and positively affected parts and consists of ten items. Its sum scores range from 0 to 30, with higher scores indicating higher degrees of depressive symptoms. A cutoff of 10 was used to identify depression individuals^28^.

CKD was based on self-reported physicians’ diagnosis ‘Have you ever been told by a doctor that you have kidney diseases?’ or personal eGFR level^29^. If an affirmative answer was given by an individual or his/her proxy respondent or eGFR < 60 mL/min per 1.73 m^2^, then the individual would be categorized as experiencing the first-time CKD in his/her life (except for tumor or cancer). Serum creatinine was measured using a rateblanked and compensated Jaffe creatinine method. Assessment of eGFR used the Chronic Kidney Disease Epidemiology Collaboration creatinine equation with adjusted coefficient of 1.1 for the Chinese population^30^.

The other covariates collected included age, sex, living place (rural vs. urban), smoking status (ever smoking vs. never smoking), drinking status (ever drinking vs. never drinking), body mass index (the weight in kilograms divided by the square of the height in meters), the presence or absence of other chronic diseases (defined by a self-reported history of dyslipidemia, diabetes, cancer, heart disease, stroke, asthma, lung disease, liver disease, digestive disease, and memory problem). “Ever smoking” means that the respondent reports smoking at some point, and “never smoking” means that the respondent reports never having smoked. “Ever drinking” means that the respondent reports having had an alcoholic beverage in the past, and “never drinking” means that the respondent reports not having any alcoholic beverage in the past. Moreover, dyslipidemia (defined by a history of dyslipidemia, or triglyceride ≥ 2.26 mmol/L, or total cholesterol ≥ 6.22 mmol/L, or high-density lipoprotein cholesterol < 1.04 mmol/L, or low-density lipoprotein cholesterol ≥ 4.14 mmol/L), diabetes (defined by a history of diabetes, or fasting glucose ≥ 6.1 mmol/L, or non-fasting glucose ≥ 7.8 mmol/L)^31^.

### Statistical analysis

Participants’ baseline characteristics are presented as percentages for categorical variables, as the means with standard deviation for normally distributed variables and as medians with interquartile range for non-normally distributed variables. Demographic and clinical characteristics were compared between groups by Student t tests, or Wilcoxon rank-sum tests for continuous variables and χ2 test for categorical variables as appropriate. Multivariate logistic regression models were used to calculate odds ratios (ORs) and 95% confidence intervals (95% CIs) of the relationships between depression and CKD. Potential covariates, such as age, sex, living place, education level, smoking, drinking, body mass index, systolic blood pressure, and medical history (dyslipidemia, diabetes, cancer, heart disease, stroke, asthma, lung disease, liver disease, digestive disease, and memory problem) were included in the multivariable models. Furthermore, subjects were further divided into three groups according to CESD-10 scores: < 10; 10–12; ≥ 12 in study I, and according to eGFR level and self-reported kidney diseases: eGFR ≥ 90 mL/min per 1.73 m^2^; 60 ≤ eGFR < 90 mL/min per 1.73 m^2^; eGFR < 60 mL/min per 1.73 m^2^ or with reported kidney diseases in study II. The subgroup analyses were further performed to evaluate the association between depression and CKD according to sex, age, living place, smoking, drinking, education level, history of hypertension and diabetes. In sensitivity analyses, we further adjusted baseline eGFR level in study I and baseline CESD-10 scores in study II based on multivariable models. Two tailed *P* < 0.05 was considered to be statistically significant. All statistical analyses were conducted using SAS statistical software (version 9.4, Cary, NC).

## Results

### Study I: Association between baseline depression with subsequent CKD

In study I, a total of 7637 participants (3542 men and 4095 women) were included in the analysis, and the average age was 57.59 ± 8.23 years. Of them, 4888 (66.34%) participants were defined as non-depression individuals, and 2749(33.66%) participants were defined as depression. Compared to individuals without depression, participants with depression tended to be older and female. The baseline characteristics such as living place, education level, history of dyslipidemia, smoking, drinking, SBP and DBP were significantly different between two subgroups (Table 1).

**Table 1:** Characteristics of the study population at baseline in study I and study II.

Characteristics	Study I (N = 7637)			Study II(N = 6337)		
	Without depression	With depression	*P* value	Without CKD	With CKD	*P* value
No. of subjects	4888	2749		5189	1148	
Age, years	57.17 ± 8.17	58.33 ± 8.28	< 0.001	56.97 ± 8.36	63.83 ± 10.20	< 0.001
**Sex, n (%)**						
Male	2541(51.98)	1001(36.41)	< .0.001	2716(52.34)	602(52.44)	0.952
Female	2347(48.02)	1748(63.59)		2473(47.66)	546(47.56)	
**Living place, n (%)**						
Urban	1848 (37.81)	797(28.99)	< .0.001	1892(36.46)	508(44.25)	
Rural	3040(62.19)	1952(71.01)		3297(63.54)	640(55.75)	
**Education level, n (%)**						
Below primary school	1138(23.28)	985(35.83)	< 0.001	1263(24.34)	314(27.35)	< 0.001
Primary school	1945(39.79)	1154(41.98)		2004(38.62)	484(42.16)	
Middle school	1195(24.45)	444(16.15)		1252(24.13)	213(18.55)	
High school or above	610(12.48)	166(5.49)		670(12.91)	137(11.97)	
**Medical history**						
Hypertension, n (%)	1095(22.40)	575(20.92)	0.132	1162(22.39)	275(23.95)	0.253
Dyslipidemia, n (%)	451(9.23)	305(11.09)	0.009	463(8.92)	126(10.98)	0.030
Diabetes mellitus, n (%)	279(5.71)	183(6.66)	0.095	285(5.49)	88(6.05)	0.005
Cancer, n (%)	41(0.84)	23(0.84)	0.992	51(0.98)	13(1.13)	0.647
Smoking, n (%)	2028(41.50)	932(33.90)	< 0.001	2143(41.46)	491(43.07)	0.318
Drinking, n (%)	1978(40.47)	977(35.54)	< 0.001	2136(41.46)	479(41.72)	0.727
BMI (kg/m^2^)	23.40(21.50–25.51)	23.40(20.84–25.30)	0.339	23.40(21.57–25.28)	23.40(21.67–25.68)	0.083
SBP, mmHg	129.45 ± 19.62	128.37 ± 19.83	0.021	128.98 ± 19.26	133.30 ± 20.47	< 0.001
DBP, mmHg	76.15 ± 11.51	75.03 ± 11.71	< 0.001	75.94 ± 10.87	75.50 ± 11.39	0.221

BMI: body mass index; SBP: systolic blood pressure; DBP: diastolic blood pressure;

Continuous variables are expressed as mean ± standard deviation, or as median (interquartile range). Categorical variables are expressed as frequency (percent).

^†^CKD eGFR < 90 mL/min per 1.73 m^2^ or with reported kidney diseases.

During 2011 to 2015 (Wave 1 to Wave 3), a total of 301 (6.16%) respondents experienced CKD in the participants without depression, and 233 (8.48%) respondents experienced CKD in the participants with depression. Participants with depression had higher risk of CKD than those without depression with crude ORs (95% CIs) of 1.30(1.02–1.66). After multivariable adjustment, the association persisted significant, with corresponding ORs (95% CIs) was 1.38(1.08–1.76) (Table 2). When individuals were divided into three groups, participants with higher CESD-10 score (≥ 12) had higher risk of developing CKD (ORs = 1.24; 95%CIs 1.02–1.69) compared with those with CESD-10 score < 10 (Table 3). In subgroup analysis, the significant associations of depression with risk of CKD were observed in almost all subgroups, and significant interactions between depression and any of these interested variables on CKD were observed in diabetes subgroups (Table 4). When baseline eGFR levels were further adjusted in sensitivity analyses, the association between depression and CKD remained significant (Table S1).

**Table 2:** Bidirectional association between depression and kidney disease.

	Crude		Age and sex- adjusted		Multivariable-adjusted*	
	OR (95%CI)	*P* value	OR (95%CI)	*P* value	OR (95%CI)	*P* value
**Study I**						
Without depression	1.00(Ref)		1.00(Ref)		1.00(Ref)	
With depression	1.24(1.09–1.41)	0.001	1.19(1.04–1.36)	0.008	1.22(1.06–1.39)	0.004
**Study II**						
Without CKD†	1.00(Ref)		1.00(Ref)		1.00(Ref)	
With CKD	1.31(1.15–1.69)	0.016	1.26(1.09–1.55)	0.026	1.18(1.04–1.46)	0.016

* Multivariable-adjusted for age, sex, living place, education level, smoking, drinking, body mass index, systolic blood pressure, and medical history (dyslipidemia, diabetes, cancer, heart disease, stroke, asthma, lung disease, liver disease, digestive disease, and memory problem).

^†^ CKD eGFR < 90 mL/min per 1.73 m^2^ or with reported kidney diseases.

**Table 3:** Sensitivity analyses of the association between depression and kidney disease.

	Crude		Age and sex- adjusted		Multivariable-adjusted*	
	OR (95%CI)	*P* trend	OR (95%CI)	*P* trend	OR (95%CI)	*P* trend
**Study I**						
CESD-10 score < 10	1.00(Ref)		1.00(Ref)		1.00(Ref)	
10 ≤ CESD-10 score < 12	0.99(0.79–1.25)		1.00(0.79–1.25)		1.04(0.90–1.21)	
CESD-10 score ≥ 12	1.33(1.16–1.53)	< 0.001	1.26(1.09–1.47)	< 0.001	1.24(1.02–1.69)	0.003
**Study II**						
eGFR ≥ 90 mL/min per 1.73 m^2^	1.00(Ref)		1.00(Ref)		1.00(Ref)	
60 ≤ eGFR < 90 mL/min per 1.73 m^2^	1.03(0.86–1.23)		1.07(0.88–1.29)		1.19(0.97–1.45)	
eGFR < 60 mL/min per 1.73 m^2^ or with reported CKD	1.31(1.02–1.66)	0.046	1.36(1.07–1.74)	0.020	1.32(1.02–1.71)	0.013

* Multivariable-adjusted for Multivariable-adjusted for age, sex, education level, living place, smoking, drinking, body mass index, systolic blood pressure, and medical history (dyslipidemia, diabetes, cancer, heart disease, stroke, asthma, lung disease, liver disease, digestive disease, and memory problem).

^†^CKD eGFR < 90 mL/min per 1.73 m^2^ or with reported kidney diseases.

**Table 4:** Subgroup analysis of the bidirectional association between depression and kidney disease.

Characteristics	Study I (N = 7367)				Study II(N = 7367)			
	Without depression	With depression	*P* value	*P*-_interaction_	Without CKD	With CKD †	*P* value	*P*-_interaction_
**Sex**								
Male	1.00(Ref)	1.22(1.01–1.49)	0.049	0.373	1.00(Ref)	1.21(0.96–1.54)	0.114	0.084
Female	1.00(Ref)	1.32(1.10–1.59)	0.004		1.00(Ref)	1.17(0.94–1.45)	0.174	
**Age, years**								
< 60	1.00(Ref)	1.31(1.14–1.50)	0.001	0.051	1.00(Ref)	1.29(1.02–1.63)	0.037	0.015
≥ 60	1.00(Ref)	1.27(1.05–1.53)	0.012		1.00(Ref)	1.08(0.88–1.33)	0.469	
**Living place**								
Urban	1.00(Ref)	1.26(1.10–1.44)	0.001	0.514	1.00(Ref)	1.10(0.95–1.28)	0.211	0.060
Rural	1.00(Ref)	1.11(0.88–1.40)	0.394		1.00(Ref)	1.20(1.03–1.54)	0.036	
**Hypertension**								
Urban	1.00(Ref)	1.32(1.13–1.54)	0.002	0.318	1.00(Ref)	1.07(0.89–1.27)	0.473	0.341
Rural	1.00(Ref)	1.14(0.85–1.54)	0.373		1.00(Ref)	1.43(1.05–1.96)	0.024	
**Smoking**								
No	1.00(Ref)	1.33(1.11–1.58)	0.002	0.201	1.00(Ref)	1.25(1.04–1.39)	0.044	0.500
Yes	1.00(Ref)	1.18(0.95–1.47)	0.133		1.00(Ref)	1.18(.92–1.50)	0.187	
**Drinking**								
No	1.00(Ref)	1.28(1.02–1.61)	0.028	0.972	1.00(Ref)	1.09(0.89–1.32)	0.412	0.616
Yes	1.00(Ref)	1.26(1.06–1.50)	0.009		1.00(Ref)	1.25(1.07–1.60)	0.038	
**Diabetes**								
No	1.00(Ref)	1.26(1.06–1.50)	0.009	< 0.001	1.00(Ref)	1.16(1.01–1.36)	0.045	0.987
Yes	1.00(Ref)	1.28(1.03–1.61)	0.028		1.00(Ref)	0.91(0.51–1.64)	0.761	
**Education level**								
Below primary school	1.00(Ref)	1.27(1.06–1.53)	0.009	0.373	1.00(Ref)	1.16(0.94–1.42)	0.162	0.400
Primary school	1.00(Ref)	1.30(1.06–1.60)	0.013		1.00(Ref)	1.30(1.04–1.62)	0.020	
Middle school	1.00(Ref)	1.24(0.94–1.65)	0.128		1.00(Ref)	1.29(1.04–1.61)		
High school or above	1.00(Ref)	1.30(1.06–1.58)	0.010		1.00(Ref)	1.22(0.98–1.52)		

In the multivariate models, confounding factors such as Multivariable-adjusted for age, sex, living place, education level, smoking, drinking, body mass index, systolic blood pressure, and medical history (dyslipidemia, diabetes, cancer, heart disease, stroke, asthma, lung disease, liver disease, digestive disease, and memory problem) were included unless the variable was used as a subgroup variable.

^†^ CKD eGFR < 90 mL/min per 1.73 m^2^ or with reported kidney diseases.

### Study II: Association between baseline depression with subsequent CKD

According to inclusion and exclusion criteria, a total of 6337 participants (3318 men and 3019 women), who were free of depression at baseline, with the average age of 58.21 ± 9.12 years were included in the analysis, including 5980 (94.37%) participants without CKD and 357 (5.63%) participants with CKD. Participants with CKD tended to be older, had higher prevalent of hypertension, dyslipidemia, smoking and drinking.

During a 4-year follow-up, there were 1333 (22.29%) subjects in the non-CKD group and 97 (27.17%) in CKD group developed depressive symptoms. Individuals with CKD had higher risk of developing depression than those without CKD, with the crude ORs (95% CIs) was 1.41(1.18–1.69). After multivariable adjustment of age, sex and other covariates, the association persisted significant, with corresponding ORs (95% CIs) was 1.48(1.23–1.78) (Table 2). When individuals were divided into three groups, the adjusted ORs (95%CIs) for developing depression in the participants with eGFR < 60 mL/min per 1.73 m^2^ or with reported CKD was 1.93 (95%CIs 1.41–2.64) times higher than individuals with eGFR ≥ 90 mL/min per 1.73 m^2^ (Table 3). In subgroup analysis, the significant associations of CKD with risk of depression were observed in almost all subgroups, and significant interactions between CKD and any of these interested variables on depression were observed in age subgroups (Table 4). Similar to Study I, when baseline CESD-10 scores were further adjusted in multivariable model in sensitivity analyses, the results between CKD and depression were not changed a lot (Table S1).

## Discussion

The present study examined bidirectional relationships between depression and CKD in a nationally representative sample of middle aged and elderly Chinese population. Our findings demonstrated that individuals with depression were associated with an increase in CKD. In the other direction, CKD patients had higher risk of subsequent depression than those without CKD. To our best knowledge, this is the first prospective longitudinal study to examine the bidirectional associations between depression and CKD, allowing for more potent validation of findings concerning the temporal association between these two diseases.

Based on this large nationwide data, our results suggested that individuals with depression had a 38% risk of CKD than those without depression. This finding was consistent with previous studies and adds to the growing body of literatures on this topic^14–16^. Furthermore, when individuals were divided into three groups according to CESD-10 score, those with CESD-10 score more than 12 had higher risk of CKD and the trend was significant, which was consistent with earlier research, meaning that the risk of CKD increased with the severity of depression^32^. Previous studies had supported a bidirectional association between inflammation and depression in chronic illness^33^, and this association is particularly relevant for patients with CKD and ESRD, in whom inflammatory levels are high^34^. Furthermore, several potential biological mechanism may explain the association between depression and CKD^35^.

Apart from the risk of depression on subsequent CKD, our findings indicated that CKD patients are at an elevated risk of developing depression than those without CKD. Furthermore, when we divided individuals into three groups, individuals with eGFR < 60 mL/min per 1.73 m^2^ or with reported CKD had higher risk of depression than those with eGFR ≥ 90 mL/min per 1.73 m^2^, and the trend was also significant. All of those results were echoing previous studies from the CHARLS^36^. Jia et al. used the Wave 1 to Wave 3 data of 3379 participants, and reported that both baseline eGFRcr and eGFRcr-cys were significantly associated with higher depression score during four-year follow up, and depression risk increased with a lower eGFR and there was a “dose–response” association^36^. Several potential mechanisms may explain this association. Depression may be a secondary psychological reaction to the development of the disease or a secondary to the complications or aversive symptoms of that disease^37^. Furthermore, the chronic medical illness has a direct pathophysiologic effect on the brain or has indirect physiologic effects^38^.

The complex interaction between depression and CKD is dynamic and multifactorial, caused by common ‘upstream’ risk factors and the biological, psychological, social or ‘downstream’ consequences of both disorders. Socioeconomic factors, immune system, inflammatory pathways, disturbances of the hypothalamic-pituitary axis, and changes in the parasympathetic and sympathetic nervous systems are particularly relevant to understanding the bidirectional association between depression and CKD^16^. Understanding these mechanisms can help prevent and treat depression and chronic kidney disease. In a randomized, double-blind, placebo-controlled trial involving 201 patients with stage 3, 4, or 5 non-dialysis-dependent CKD, treatment with sertraline compared with placebo for 12 weeks did not significantly improve depressive symptoms in patients with major depressive disorder^39^. Thus, it is of clinical interest to see whether preventions of depression in CKD patients and CKD in depression subjects would improve their mood, quality of life, and medical outcomes.

Strengths of this study include a large sample size and prospective, community-based, nationally representative study design, as well as rigorous assessment of renal function biomarkers according to standardized protocols, and prospective quantification of depressive symptoms using a validated questionnaire. Despite these advantages, there are some limitations. First, a self-report CESD-10 scale was used to define depression in present study rather than clinical diagnostic interview such as the Millon Clinical Multiaxial Inventory (MCMI) or the Symptom Checlist-90-Revised (SCL-90-R), which may be some bias in depressive symptoms diagnosis and severity.

Nevertheless, previous qualified studies demonstrated good reliability and effectiveness of CESD-10 in the Chinese population^40^. Second, CHARLS enrolled only middle-aged and older Chinese persons, and as such, we do not know whether the bidirectional relationships between depression and CKD was still significant in younger persons or another race. Third, 4-year follow-up may not be sufficient to assess the bidirectional association between depression and CKD. Fourth, some of the participants were excluded from this analysis due to incomplete outcome data or depressive symptoms measurements.

In conclusion, the present study evaluated the bidirectional association between depression and CKD. Our results indicated that baseline depression was associated with higher risk of CKD, and the onset of CKD also increases the risk of future depression. Considering our findings, more attention should be paid to prevention of CKD in depression subjects and depression in CKD patients. Furthermore, it is important to screen and treat of comorbid of depression and CKD in primary care.

## Supplementary Information

Supplementary Information.

## Data Availability

The details of the CHARLS data are available at its website (http://charls.pku.edu.cn/en). This analysis uses data or information from the Harmonized CHARLS dataset and Codebook, Version C as of April 2018 developed by the Gateway to Global Aging Data.
