# Supplementary material for: Prospective bidirectional associations between depression and chronic kidney diseases
Source: Sci Rep. 2022 Jun 28;12:10903. doi: 10.1038/s41598-022-15212-8 (PMC9240037; doi:10.1038/s41598-022-15212-8)
Supplement: Supplementary file 1 — Supplementary Information. [file 41598_2022_15212_MOESM1_ESM.docx]

**Table S1.** Bidirectional association between depression and kidney disease

|  | Crude | | Age and sex- adjusted | | Multivariable-adjusted* | | |
| --- | --- | --- | --- | --- | --- | --- | --- |
|  | OR (95%CI) | *P* value | OR (95%CI) | *P* value | OR (95%CI) | | *P* value |
| Study I | | | | | | | |
| Without depression | 1.00(Ref) |  | 1.00(Ref) |  | | 1.00(Ref) |  |
| With depression | 1.24(1.09-1.41) | 0.001 | 1.19(1.04-1.36) | 0.008 | | 1.28(1.11-1.46) | <0.001 |
| Study II | | | | | | | |
| Without CKD ^†^ | 1.00(Ref) |  | 1.00(Ref) |  | | 1.00(Ref) |  |
| With CKD ^†^ | 1.31(1.15-1.69) | 0.016 | 1.26(1.09-1.55) | 0.026 | | 1.23(1.04-1.46) | 0.016 |

* In Study I multivariable-adjusted for baseline eGFR level, age, sex, living place, smoking, drinking, body mass index, systolic blood pressure, and medical history (dyslipidemia, diabetes, cancer, heart disease, stroke, asthma, lung disease, liver disease, digestive disease, and memory problem).

* In Study II multivariable-adjusted for baseline CESD-10 scores, age, sex, living place, smoking, drinking, body mass index, systolic blood pressure, and medical history (dyslipidemia, diabetes, cancer, heart disease, stroke, asthma, lung disease, liver disease, digestive disease, and memory problem).

^†^ CKD eGFR < 60 mL/min per 1.73 m^2^ or with reported kidney diseases
